# In Vitro and In Vivo Anti-Inflammatory Effects of Sulfated Polysaccharides Isolated from the Edible Brown Seaweed, *Sargassum fulvellum*

**DOI:** 10.3390/md19050277

**Published:** 2021-05-15

**Authors:** Lei Wang, Hye-Won Yang, Ginnae Ahn, Xiaoting Fu, Jiachao Xu, Xin Gao, You-Jin Jeon

**Affiliations:** 1 College of Food Science and Engineering, Ocean University of China, Qingdao 266003, China; leiwang2021@ouc.edu.cn (L.W.); xiaotingfu@ouc.edu.cn (X.F.); xujia@ouc.edu.cn (J.X.); xingao@ouc.edu.cn (X.G.); 2 Department of Marine Life Sciences, Jeju National University, Jeju 63243, Korea; hyewon.yang@jejunu.ac.kr; 3 Department of Marine Bio Food Science, Chonnam National University, Yeosu 59626, Korea; gnahn@chonnam.ac.kr; 4 Marine Science Institute, Jeju National University, Jeju 63333, Korea

**Keywords:** seaweed, inflammation, RAW 264.7 macrophages, zebrafish

## Abstract

In the present study, the in vitro and in vivo anti-inflammatory effects of the sulfated polysaccharides isolated from *Sargassum fulvellum* (SFPS) were evaluated in lipopolysaccharide (LPS)-stimulated RAW 264.7 macrophages and zebrafish. The results indicated that SFPS improved the viability of LPS-stimulated RAW 264.7 macrophages from 80.02 to 86.80, 90.09, and 94.62% at the concentration of 25, 50, and 100 µg/mL, respectively. Also, SFPS remarkably and concentration-dependently decreased the production levels of inflammatory molecules including nitric oxide (NO), tumor necrosis factor-alpha, prostaglandin E_2_, interleukin-1 beta, and interleukin-6 in LPS-treated RAW 264.7 macrophages. In addition, SFPS significantly inhibited the expression levels of cyclooxygenase-2 and inducible nitric oxide synthase in LPS-treated RAW 264.7 macrophages. Furthermore, the in vivo test results indicated that SFPS improved the survival rate of LPS-treated zebrafish from 53.33 to 56.67, 60.00, and 70.00% at the concentration of 25, 50, and 100 µg/mL, respectively. In addition, SFPS effectively reduced cell death, reactive oxygen species, and NO levels in LPS-stimulated zebrafish. Taken together, these results suggested that SFPS possesses strong in vitro and in vivo anti-inflammatory activities, and could be used as an ingredient to develop anti-inflammatory agents in the functional food and pharmaceutical industries.

## 1. Introduction

Inflammatory response is a beneficial self-protective physiological process, which can be defined as a part of the complex biological response of the body tissues to harmful stimuli including pathogens, chemical compounds, and some food materials [1]. It is coordinated by a large range of mediators including pro-inflammatory cytokines, chemokines, and vasoactive amines, which promote structural and biochemical changes in the site of injury or infection, such as increasing the vascular permeability and migration of immune cells [2]. Overproduction of these inflammatory molecules may cause uncontrolled inflammation that leads to dysregulation of tissue healing and chronic inflammation, as well as causes or promotes the development of chronic inflammation-related diseases such as rheumatoid arthritis, strokes, atherosclerosis, and cancers [3,4]. A compound that could inhibit the production of inflammatory molecules may have the potential to be developed as an anti-inflammatory agent in the pharmaceutical industry.

*Sargassum fulvellum* is a popular edible seaweed, which has been consumed as an herb medicine, food material, and food additive in Asian countries for a long time. *S. fulvellum* contains various bioactive compounds such as carbohydrates, proteins, pigments, lipids, and phenolic compounds [5,6]. These compounds possess numerous bioactivities such as antioxidant, hepatoprotective, anti-atopic dermatitis, neuroprotective, anti-coagulant, antipyretic, analgesic, and anti-inflammatory effects [5,7,8,9,10]. Kang et al. investigated the inhibitory effect of the ethanol extract of *S. fulvellum* (SFEE) and the compound (grasshopper ketone) isolated from SFEE on atopic dermatitis [9]. The results indicated that SFEE and grasshopper ketone effectively decreased the severity of skin dermatitis and suppressed the serum levels of total immunoglobulin E (IgE) and pro-inflammatory cytokines including tumor necrosis factor (TNF)-α and interleukin (IL)-4 in mice [9]. Kim et al. evaluated the anti-inflammatory effects of grasshopper ketone on lipopolysaccharides (LPS)-induced inflammatory responses in RAW 264.7 macrophages [10]. The results demonstrated that grasshopper ketone significantly inhibited LPS-induced production of nitric oxide (NO) and pro-inflammatory cytokines including TNF-α, IL-6, and IL-1β, as well as inhibition of cyclooxygenase-2 (COX-2) and inducible nitric oxide synthase (iNOS) proteins expression in RAW 264.7 cells [10].

In the previous studies, we evaluated antioxidant activities of enzyme-assisted extracts of *S. fulvellum* [11]. The results indicated that the Celluclast-assisted extract of *S. fulvellum* possessed high carbohydrate content and showed strong antioxidant effect [11]. Furthermore, we prepared the sulfated polysaccharides from the Celluclast-assisted extract of *S. fulvellum* (SFPS), and investigated the in vitro and in vivo antioxidant activities of SFPS [5]. The results suggested that SFPS remarkably suppressed 2,2′-azobis(2-methylpropionamidine) dihydrochloride-induced oxidative stress in vitro in Vero cells and in vivo in zebrafish [5]. In order to further evaluate the bioactivities of SFPS, in the present study, we investigated the anti-inflammatory effect of SFPS in LPS-stimulated RAW 264.7 cells and zebrafish.

## 2. Results and Discussion

### 2.1. SFPS Suppresses Cytotoxicity and Inflammatory Molecules Production in LPS-Induced RAW 264.7 Macrophages

Sulfated polysaccharides isolated from seaweeds have been reported to possess various bioactivities [12,13,14,15,16,17]. In particular, seaweed-derived sulfated polysaccharides possess potent immunostimulatory and anti-inflammatory effects [12,17]. Our previous studies have investigated the anti-inflammatory effects of sulfated polysaccharides isolated from different seaweeds including *Chnoospora minima*, *Sargassum horneri*, *Saccharina japonica*, *Hizikia fusiforme*, and *Padina commersonii* [3,4,18,19,20]. The results indicated that these sulfated polysaccharides possess strong anti-inflammatory activities. These results confirmed the anti-inflammatory potential of seaweed-derived sulfated polysaccharides. Therefore, in the present study, we investigated the anti-inflammatory activity of SFPS.

Macrophages play a critical role in inflammatory responses via producing inflammatory mediators such as TNF-α, NO, IL-1β, and IL-6. The abnormal production of these inflammatory mediators is pivotal to the progression of inflammatory responses [18]. LPS is a component of the cell wall in gram-negative bacteria, which stimulates inflammatory responses in macrophages. Thus, in the present study, LPS-stimulated RAW 264.7 cells were used as an in vitro model to investigate the anti-inflammatory activity of SFPS.

**Figure 1:**
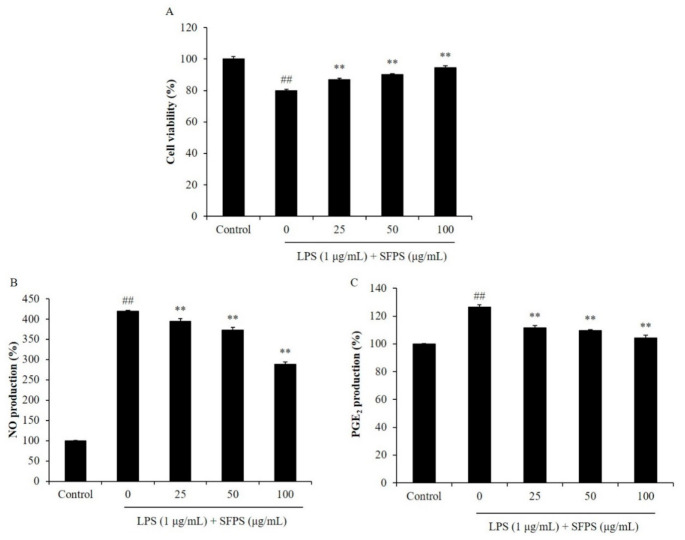
Effect of SFPS on LPS-induced cytotoxicity and the production levels of NO and PGE_2_ in RAW 264.7 cells. (**A**) The viability of LPS-stimulated RAW 264.7 cells; the production levels of NO (**B**) and PGE_2_ (**C**) in LPS-stimulated RAW 264.7 cells. RAW 264.7 cells were treated with different concentrations of SFPS (25, 50, and 100 μg/mL) and stimulated with LPS (1 μg/mL) for 24 h. The levels of PGE_2_ and NO were analyzed by ELISA and Griess assay, respectively. The cell viability was evaluated by MTT assay. The experiments were conducted in triplicate and data are expressed as mean ± SE. ** *p* < 0.01 as compared to LPS-treated group and ^##^ *p* < 0.01 as compared to control group.

In the present study, the effect of SFPS on LPS-induced cytotoxicity and the production of inflammatory molecules were evaluated. As Figure 1 shows, the viability of LPS-treated RAW 264.7 cells was significantly decreased. But, the productions of NO and PGE_2_ were increased compared to the non-treated cells. However, SFPS remarkably improved the viability of LPS-treated RAW 264.7 cells and reduced the production of NO and PGE_2_ (Figure 1). Both effects were concentration-dependent. LPS reduced the viability of RAW 264.7 cells to 80.02% compared to non-treated cells (100%). However, the viabilities of the cells treated with 25, 50, and 100 µg/mL of SFPS were improved to 86.80, 90.09, and 94.62%, respectively (Figure 1A). In addition, LPS increased 3.19 folds NO production in RAW 264.7 cells. But, SFPS decreased 0.25, 0.46, and 1.3 folds (7.8, 14.42, and 40.75%) NO production in LPS-stimulated RAW 264.7 cells at the concentration of 25, 50, and 100 µg/mL, respectively (Figure 1B). Previous study has evaluated the anti-inflammatory effect of sulfated polysaccharides isolated from *Hizikia fusiforme* (HFPS) [3]. The results indicated that HFPS increased the viabilities of LPS-treated RAW 264.7 cells from 83.89 to 88.76, 94.50, and 98.52% at the concentration of 25, 50, and 100 µg/mL, respectively [3]. Also, HFPS reduced 6.3, 20.87, and 24.87% of LPS-induced NO production in RAW 264.7 cells at the concentration of 25, 50, and 100 µg/mL, respectively [3]. These results indicated that HFPS possesses stronger anti-inflammatory activity than SFPS. Previous reports suggested that bioactivities of sulfated polysaccharides were related to their sulfate and fucose contents [3,4,5]. The chemical component analysis results displayed that the sulfate and fucose contents are 53.53% and 7.78% in HFPS, and 26.75% and 1.01% in SFPS, respectively [3]. Thus, we thought that SFPS possesses anti-inflammatory potential is owing to its sulfate and fucose contents. Moreover, the anti-inflammatory effect of SFPS is not stronger than HFPS may because it contains lower sulfate and fucose contents than HFPS.

### 2.2. The Inhibitory Effect of SFPS against Pro-Inflammatory Cytokines, iNOS, and COX-2 Expression in LPS-Stimulated RAW 264.7 Macrophages

**Figure 2:**
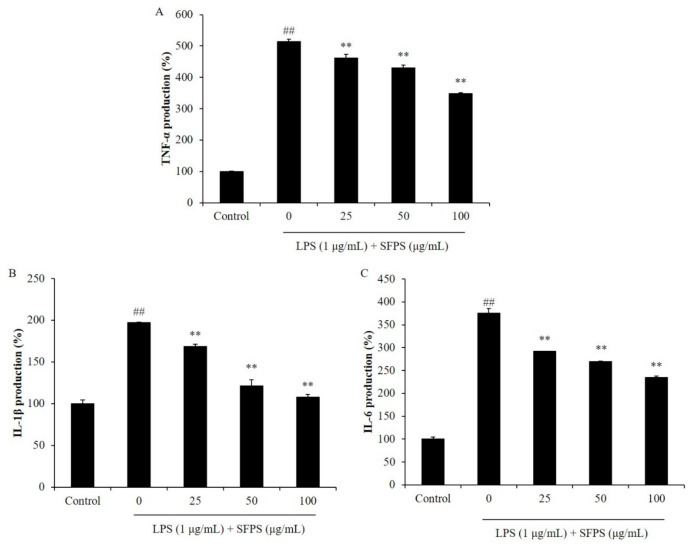
Effect of SFPS on the production levels of pro-inflammatory cytokines in LPS-stimulated RAW 264.7 cells. (**A**) Production of TNF-α; (**B**) production of IL-1β; (**C**) production of IL-6. RAW 264.7 cells were treated with different concentrations of SFPS (25, 50, and 100 μg/mL) and stimulated with LPS (1 μg/mL) for 24 h. The levels of pro-inflammatory cytokines were analyzed by ELISA. The experiments were conducted in triplicate and data are expressed as mean ± SE. ** *p* < 0.01 as compared to LPS-treated group and ^##^ *p* < 0.01 as compared to control group.

**Figure 3:**
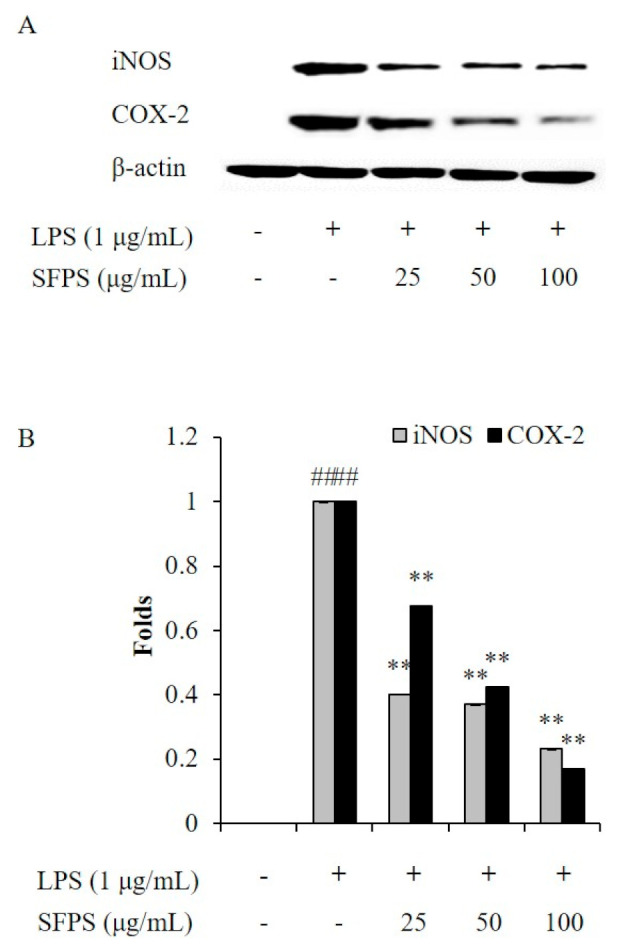
Effect of SFPS on the expression levels of iNOS and COX-2 in LPS-stimulated RAW 264.7 cells. (**A**) Inhibitory effect of SFPS on iNOS and COX-2 expression, and (**B**) relative amounts of iNOS and COX-2. RAW 264.7 cells were treated with different concentrations of SFPS (25, 50, and 100 μg/mL) and stimulated with LPS (1 μg/mL) for 24 h. The levels of iNOS and COX-2 were analyzed by western blot assay. The relative amounts of iNOS and COX-2 were compared with β-actin. The experiments were conducted in triplicate and data are expressed as mean ± SE. ** *p* < 0.01 as compared to LPS-treated group and ^##^ *p* < 0.01 as compared to control group.

Previous reports indicated that overproduction of NO is mainly caused by iNOS, which is not expressed under normal condition but expressed after the generation of pro-inflammatory cytokines [21,22,23]. PGE_2_ is an inflammatory molecule produced in macrophages and involved in vasodilation, pain and fever in the early stages of the inflammatory response [23]. COX-2 plays a major role in the expression of PGE_2_. Previous studies suggested that iNOS and COX-2 inhibitor reduced the production of NO and PGE_2_, consequently attenuated inflammation [1,18]. In order to further investigate the anti-inflammatory effect of SFPS, the effect of SFPS on LPS-stimulated the productions of pro-inflammatory cytokines, iNOS, and COX-2 in RAW 264.7 cells was evaluated. The results indicated that SFPS effectively suppressed productions of pro-inflammatory cytokines including TNF-α, IL-1β, and IL-6 in LPS-stimulated RAW 264.7 cells in a concentration-dependent manner (Figure 2). Furthermore, LPS significantly stimulated the expressions of iNOS and COX-2 in RAW 264.7 cells (Figure 3). However, the expressions of iNOS and COX-2 were remarkably and concentration-dependently reduced in SFPS-treated RAW 264.7 cells (Figure 3). These results displayed that SFPS suppressed LPS-induced inflammatory response via reducing the productions of NO and PGE_2_ by inhibiting the expression of iNOS and COX-2 through decreasing the levels of pro-inflammatory cytokines in RAW 264.7 cells.

### 2.3. SFPS Improves LPS-Induced Inflammatory Response in Zebrafish

Zebrafish is a popular in vivo model used in pharmacological, biological, and toxicological researches, due to their advantages, such as its signal transduction pathway is basically similar to humans, as well as its genome, biological structure, and physiological function similar to mammals [1,22]. Zebrafish stimulated with LPS was used to investigate the in vivo anti-inflammatory activities of seaweed-derived compounds including sulfated polysaccharides in our previous studies [3,4,18,19,20,24,25]. Thus, in the present study, LPS-stimulated zebrafish was selected as the in vivo model to evaluate the anti-inflammatory effect of SFPS.

**Figure 4:**
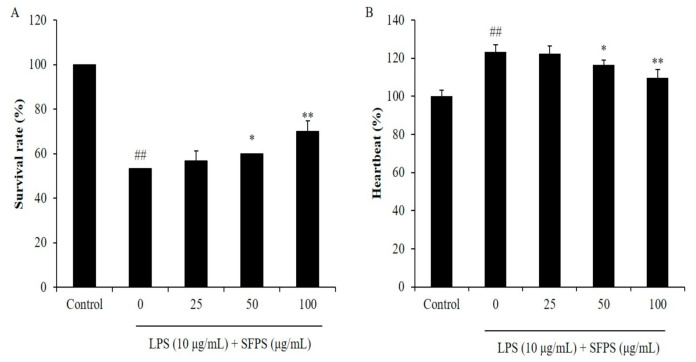
Survival rate and heartbeat of zebrafish after being treated with SFPS or/and with LPS. (**A**) Survival rate of zebrafish, and (**B**) heartbeat of zebrafish. At 7–9 hpf, the zebrafish embryos were treated with different concentration of SFPS (25, 50, and 100 μg/mL) and stimulated with LPS (10 μg/mL) until 24 hpf. The heartbeat rate was measured at 2 dpf and the survival rate was measured at 3 dpf. The experiments were conducted in triplicate and data are expressed as mean ± SE. * *p* < 0.05, ** *p* < 0.01 as compared to LPS-treated group and ^##^ *p* < 0.01 as compared to control group.

**Figure 5:**
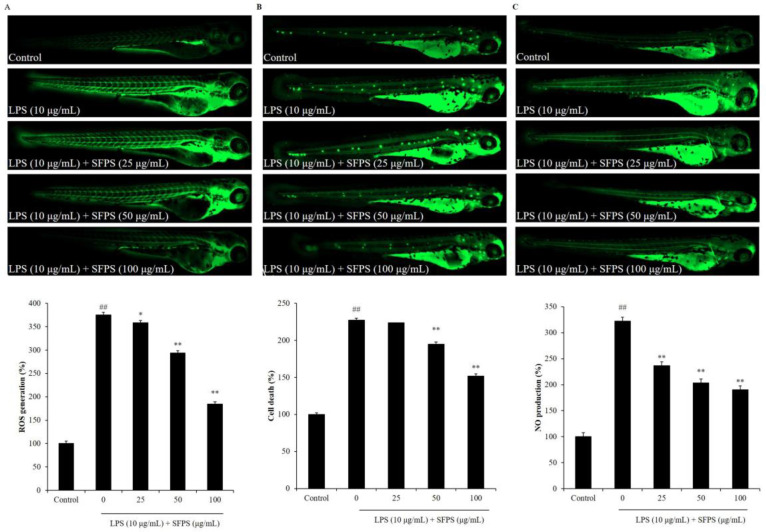
Effect of SFPS on inflammatory response in LPS-induced zebrafish. (**A**) ROS levels of LPS-stimulated zebrafish, (**B**) cell death of LPS-stimulated zebrafish, and (**C**) NO production in LPS-stimulated zebrafish. At 7–9 hpf, the zebrafish embryos were treated with different concentrations of SFPS (25, 50, and 100 μg/mL) and stimulated with LPS (10 μg/mL) until 24 hpf. At 3 dpf, the levels of ROS, cell death, and NO in LPS-stimulated zebrafish were measured in live zebrafish larvae using DCFH2-DA, acridine orange, and DAF-FM-DA staining, respectively. The relative amounts of ROS, cell death, and NO of zebrafish were measured using Image J software. The experiments were conducted in triplicate and data are expressed as the mean ± SE. * *p* < 0.05, ** *p* < 0.01 as compared to the LPS-treated group and ^##^ *p* < 0.01 as compared to control group.

In the present study, the effect of SFPS on survival rate decreasing, heartbeat disorder, and inflammatory response in LPS-stimulated zebrafish was investigated. As shown in Figure 4A, the survival rate of LPS-treated zebrafish was significantly decreased. However, the survival rates of zebrafish treated with SFPS were remarkably increased in a dose-dependent manner (Figure 4A). In addition, LPS significantly stimulated heartbeat disorder, but the heartbeat disorder of zebrafish was effectively improved by SFPS treatment. Furthermore, the levels of reactive oxygen species (ROS), cell death, and NO in LPS-treated zebrafish were significantly increased compared to the zebrafish non-treated with LPS (Figure 5). Whereas, the levels of ROS, cell death, and NO of LPS-treated zebrafish were effectively reduced by SFPS in a dose-dependent manner (Figure 5). These results demonstrated that SFPS could effective suppresses LPS-stimulated inflammatory response in vivo in zebrafish.

In summary, in the present study, the in vitro and in vivo anti-inflammatory effects of SFPS were investigated in LPS-stimulated RAW 264.7 cells and zebrafish. The results displayed that SFPS suppressed LPS-induced inflammatory response displayed in reducing the productions of NO and PGE_2_; inhibiting the expression of iNOS and COX-2; and decreasing the levels of pro-inflammatory cytokines in RAW 264.7 cells. Furthermore, the in vivo test indicated that SFPS significantly improved the survival rate; suppressed heartbeat disorder; and reduced the levels of ROS, cell death, and NO in LPS-stimulated zebrafish. These results demonstrated that SFPS possesses potent in vitro and in vivo anti-inflammatory effects.

## 3. Materials and Methods

### 3.1. Reagents and Chemicals

3-(4,5-Dimethylthiazol-2-yl)-2,5-diphenyltetrazolium bromide (MTT), 2′,7′-dichlorodihydrofluorescein diacetate (DCFH2-DA), 3-Amino-4-aminomethyl-2′,7′-difluorescein diacetate (DAF-FM-DA), Celluclast (Sigma, St. Louis, MO, USA, ≥700 units/g), LPS, and dimethyl sulfoxide (DMSO) were procured from Sigma-Aldrich Co. (St. Louis, MO, USA). Fetal bovine serum (FBS), trypsin-EDTA, Dulbecco’s modified Eagle’s medium (DMEM), and penicillin-streptomycin (p/s) were purchased from Gibco-BRL (Grand Island, NY, USA). Enzyme-linked immunosorbent assay (ELISA) kits used for the analysis of IL-1β, PGE_2_, IL-6, and TNF-α levels were purchased from R&D Systems Inc. (Minneapolis, MN, USA). COX-2, β-actin, iNOS, and anti-rabbit antibodies were purchased from Thermo Scientific (Waltham, MA, USA). All other chemicals used in this study were analytical grade.

### 3.2. Preparation of SFPS

SFPS was prepared as described in the previous study [5]. The lyophilized *S. fulvellum* powder (10 g) were hydrolyzed by Celluclast (1 mL) at 50 °C for 24 h with agitation (pH 4.5, 1 L). After reaction, the Celluclast extract of *S. fulvellum* concentrated after filtration was precipitated by adding 95% ethanol (2 L) and then the sulfated polysaccharides of *S. fulvellum* (SFPS) with an extraction yield as 23.62% were obtained. SFPS contains 1.01% sulfate and 73.54% polysaccharides. Taken together, SFPS contains 74.55% sulfated polysaccharides. The monosaccharides of the SFPS were determined by high-performance anion-exchange chromatography with pulsed amperometric detection. The amount of each monosaccharide was calculated according to the standard curve and displayed by their relative ratios. The results indicated that SFPS are composed of 26.75% fucose, 33.77% galactose, 7.11% glucose, and 31.77% xylose.

According to the previous study, 100 μg/mL of SFPS is the maximum concentration for cells and zebrafish [5]. Thus, in the present study, 100 μg/mL was determined as the maximum concentration treated to RAW 264.7 cells and zebrafish. SFPS was dissolved in 1X PBS and treat to RAW 264.7 cells and zebrafish.

### 3.3. Cell Culture

RAW 264.7 macrophages were obtained from the American Type Culture Collection (RAW 264.7 cells, TIB-71^TM^, Manassas, VA, USA) were cultured in DMEM (10% FBS and 1% P/S) and seeded at a concentration of 1 × 10^5^ cells/mL for the experiments.

### 3.4. Measurement of NO Production and Cell Viability

RAW 264.7 cells were seeded in 24-well plate and incubated for 24 h. Cells were treated with 25, 50, and 100 µg/mL of SFPS. After 1 h, the SFPS-treated cells were stimulated with LPS (1 µg/mL) and incubated for 24 h. After incubation, the viability of LPS-stimulated RAW 264.7 cells was determined by a MTT assay, and the production of NO was measured by a Griess assay according to protocols described previously [3,26,27].

### 3.5. Enzyme-Linked Immunosorbent Assay

RAW 264.7 cells were seeded in 24-well plate and incubated for 24 h. Cells were treated with SFPS and stimulated with LPS. After 24 h incubation, the cell culture media was collected and the production levels of PGE_2_, IL-1β, IL-6, and TNF-α were evaluated using ELISA kits following the manufacturer’s instructions.

### 3.6. Western Blot Analysis

RAW 264.7 cells were treated with SFPS and stimulated with LPS. Following an incubation period of 24 h, the LPS-stimulated RAW 264.7 cells were harvested and lysed. Western blot analysis was performed according to the protocol described previously [3,5].

### 3.7. Application of SFPS and LPS to Zebrafish

Adult zebrafish were maintained as described in a previous study [5]. Approximately 7–9 h post-fertilization (hpf), the embryos (15 embryos/well in a 12-well plate) were incubated with the embryo media containing SFPS with a final concentration of 25, 50, and 100 μg/mL. After 1 h incubation, the embryos were stimulated with LPS (10 μg/mL) until 24 hpf. The survival rate was measured at 3 days post-fertilization (dpf) by counting the surviving fish and these were used for further analysis. Approval for the zebrafish experiments was obtained from the Animal Care and Use Committee of the Jeju National University (Approval No. 2019-O-0074).

### 3.8. Measurement of Heartbeat, ROS Generation, NO Production, and Cell Death in Zebrafish

The heartbeat of LPS-stimulated zebrafish was measured at 2 dpf according to the protocol described by Kim et al. [28]. The levels of ROS, cell death, and NO in LPS-stimulated zebrafish were measured in live zebrafish larvae using DCFH2-DA, acridine orange, and DAF-FM-DA staining, respectively, base on the methods reported previously [1,25].

### 3.9. Statistical Analysis

The experiments were conducted in triplicate and the data are expressed as mean ± standard error. One-way ANOVA was used to compare the mean values of each treatment by SPSS 20.0 software (IBM, New York, NY, USA). Significant differences between the means were estimated employing the Tukey test.

## 4. Conclusions

This study investigated the in vitro and in vivo anti-inflammatory effects of the sulfated polysaccharides of the edible seaweed *S. fulvellum* (SFPS). The results indicated that SFPS effectively suppressed inflammatory response stimulated by LPS in vitro in RAW 264.7 cells and in vivo in zebrafish. Present results suggested that SFPS is an ideal anti-inflammatory ingredient in functional food and pharmaceutical industries.

## Data Availability

Not applicable.
